# Physical and Chemical Properties Characterization of 3D-Printed Substrates Loaded with Copper-Nickel Nanowires

**DOI:** 10.3390/polym12112680

**Published:** 2020-11-13

**Authors:** Ely Dannier V-Niño, Quentin Lonne, Andrés Díaz Lantada, Enrique Mejía-Ospino, Hugo Armando Estupiñán Durán, Rafael Cabanzo Hernández, Gustavo Ramírez-Caballero, José Luis Endrino

**Affiliations:** 1 BCMaterials, Basque Center for Materials, Applications and Nanostructures, UPV/EHU Science Park, 48940 Leioa, Spain; 2 Materials Science and Technology Research Group, Foundation of Researchers in Science and Technology of Materials, 680003 Bucaramanga, Colombia; 3 School of Aerospace, Transport and Manufacturing, Cranfield University, Bedfordshire MK43 0AL, UK; quentin.lonne@gmail.com; 4 Departamento de Ingeniería Mecánica, Universidad Politécnica de Madrid, 28006 Madrid, Spain; adiaz@etsii.upm.es; 5 Escuelas de Química, Física e Ingeniería Química, Universidad Industrial de Santander, 680002 Bucaramanga, Colombia; emejia@uis.edu.co (E.M.-O.); rcabanzo@uis.edu.co (R.C.H.); gusramca@uis.edu.co (G.R.-C.); 6 Departamento de Materiales y Minerales, Universidad Nacional de Colombia, 050034 Medellín, Colombia; haestupinand@unal.edu.co; 7 IKERBASQUE, Basque Foundation for Science, 48013 Bilbao, Spain

**Keywords:** nanofillers, bulk-functionalization, photopolymer resin, additive manufacturing, stereolithography

## Abstract

This study deals with the laser stereolithography manufacturing feasibility of copper-nickel nanowire-loaded photosensitive resins. The addition of nanowires resulted in a novel resin suitable for additive manufacturing technologies based on layer-by-layer photopolymerization. The pure and nanowire-loaded resin samples were 3D printed in a similar way. Their morphological, mechanical, thermal, and chemical properties were characterized. X-ray computed tomography revealed that 0.06 vol % of the composite resin was filled with nanowires forming randomly distributed aggregates. The increase of 57% in the storage modulus and 50% in the hardness when loading the resin with nanowire was attributed to the load transfer. Moreover, the decrease in the glass transition temperature from 57.9 °C to 52.8 °C in the polymeric matrix with nanowires evidenced a decrease in the cross-linking density, leading to a higher mobility of the polymer chains during glass transition. Consequently, this research demonstrates the successful dispersion and use of copper-nickel nanowires as a reinforcement material in a commercial resin for laser stereolithography.

## 1. Introduction

Additive manufacturing (AM) technologies, often employed for rapid prototyping (RP), directly manufacture complex three-dimensional (3D) objects layer-by-layer using the information of computer-aided design (CAD) files. Polymeric, metallic, ceramic, and even composite materials can be processed, employing a wide range of technologies, among which vat photopolymerization stands out due to the degree of precision and throughput achieved [1,2,3]. Vat photopolymerization technologies, including laser stereolithography (SLA) and digital light processing (DLP), work with a vat of photopolymerizable resin. They use the power of a laser or a light projected through a digital mask to selectively activate the polymerization of the resin in the vat, layer by layer. SLA, a laser which usually works in the ultraviolet range, is used for precisely defining the contours and infill of the polymeric layers. Both bottom-up and top-down systems exist, depending on the movement of the 3D printing bed inside the vat. The top-down equipment offers an excellent process stability, while the bottom-up set-ups stand out for their design simplicity. Sometimes however, the latter present some limitations related to the lack of adhesion of the 3D-printed part with the printing bed [1,2,3].

SLA is a 3D printing technique that can produce complex 3D structures easily, rapidly, and precisely; the SLA technique allows the production of a wide range of shapes and components and is considered as a method of reference in rapid RP [2,4,5,6,7]. It is already used to manufacture microelectromechanical systems (MEMSs) and microfluidic devices [5,6,8,9,10,11], and loading the photopolymerizable resin matrix with functional fillers opens the possibility of new composite materials and devices with tailored properties [12,13,14,15]. Several studies already present the development of functional composites with improved performance related to the manufacturing of flexible component materials [16,17,18]. Besides this, both the surface and bulk functionalization of the essential elements may lead to final devices with enhanced properties and innovative functionalities [7,8,9,10,11,12,13,14,15,19,20].

This study presents the evaluation of the physicochemical properties of substrates printed by SLA using a photosensitive resin with dispersed copper-nickel nanowires (CuNi NWs) as reinforcing agents. Additionally, the methods and materials used in the design and manufacture of the selected specimens are described. Hence, the analysis and discussion of the results obtained provide useful information regarding the potential benefits of the present approach.

## 2. Experimental Details

### 2.1. Materials and Methods

The base material used to manufacture the specimens was the commercial photoreactive resin “Clear FLGPCL 02” (Form 1+, Formlabs, Somerville, MA, USA) [10,11,15,19,21], which is a mixture of methacrylic acids esters (methacrylate oligomers and monomers) and photoinitiators, according to the manufacturer’s datasheet.

The CuNi NWs for mass-functionalization were provided by the Surface Engineering and Nanotechnology Institute (Cranfield University, Bedford, UK) in a solution of isopropyl alcohol (IPA). The total mass of this IPA/NW ink was 9.38 g, including 0.16 g of CuNi NWs. The process used to synthesize pure Cu NWs has been previously reported by some of the authors [22]. Typically, copper chloride dihydrate and nickel acetate hexahydrate were mixed and heated at 190 °C in oleylamine. The nickel ions Ni^2+^ were reduced by the oleylamine in Ni^0^, and, subsequently, Ni^0^ reduced the ${\mathrm{Cu}}^{2+}$ ions in ${\mathrm{Cu}}^{0}$ in a galvanic reaction. The nickel only had a catalytic role, and pure Cu NWs were obtained. In this study, however, the temperature of the synthesis was increased to above 200 °C to enhance the reduction kinetics of the catalytic nickel, and, hence, some nickel was incorporated into the NWs (~20 mol%). Consequently, CuNi alloy NWs were obtained instead of pure Cu NWs [22]; they were washed and stored in IPA to form an IPA/NW ink.

**Figure 1:**
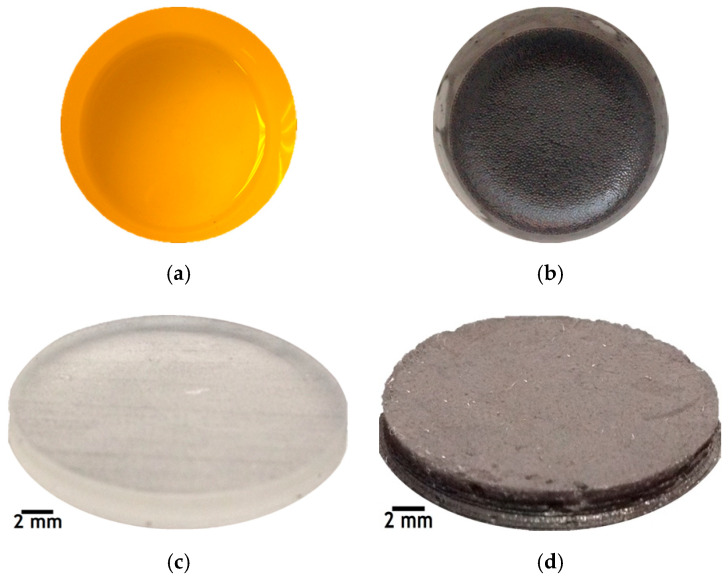
Pictures of (**a**) the pure resin, (**b**) the resin mixed with the IPA/NW ink, the SLA-printed (**c**) pure resin substrate, and (**d**) the CuNi NW-loaded substrate.

The IPA/NW ink was mixed with an incline of 30° at high speed in the MPC 004ST vacuum casting machine (SLM Solutions, Lübeck, Germany) for 30 min, and then with a magnetic stirrer bar for 90 min to 1000 rpm in the stirring hot plates model SPA 1020B (Thermolyne Corporation, Dubuque, IA, USA); Figure 1a shows the pure resin while Figure 1b shows the CuNi NW-loaded resin.

Cylindrical substrates with a diameter of 21.3 mm and a thickness of 3 mm were designed using the FreeCAD v0.16 software and the CAD designs were exported to Standard Tessellation Language/STereoLithography (STL) format, which is a standard file format for additive manufacturing technologies and 3D printers [19]. The fabrication of the substrates with and without CuNi NWs was then carried out using the SLA technique with a Formlabs Form 1+ printer [7,15,19,21]. It followed a bottom-up approach, starting with the printing bed completely immersed in the resin vat, and used a laser to photopolymerize the resin layer-by-layer from below, through the transparent bottom of the vat [3,15,19]. Three substrates of pure resin (Figure 1c) and 3 substrates of CuNi NW-loaded resin (Figure 1d) were SLA-printed, with a resolution of 100 μm per layer and a total printing time of 34 min [19].

### 2.2. Characterization Techniques

The surface morphology and elemental composition of the SLA-printed, CuNi NW-loaded substrates were analyzed by scanning electron microscopy (SEM) in the microscope thermionic emission ZEISS EVO MA10 (ZEISS, Oberkochen, Germany); from the images obtained by secondary electrons and backscattered electrons, the elements’ identification and distribution on the image were acquired by electrons and energy dispersive spectroscopy (EDS) with the detector Oxford X-act (Oxford Instruments, Abingdon, UK) coupled with SEM. Additionally, the 3D distribution of the CuNi NWs in the polymeric matrix was performed with a computed tomography (CT) system XT H 160 CT SCAN (Nikon, Tokyo, Japan); it used an energy beam of 150 kV and 50 μA, and the working distance between the specimen and the detector was set to optimize the phase contrast between the NWs and the polymeric matrix. The exposure time was 500 ms, where four frames (each one of 540 projections) were collected by radiography. Moreover, the voxel size was fixed at 10 μm, which was adequate to image the selected region.

The mechanical properties were characterized by nano-indentation testing with a Berkovich-type indenter, a force of 10 mN, and a 10 s creep in the IBIS-Authority nano-indentation system (Fischer-Cripps Laboratories Pty Limited, Sydney, Australia). The storage moduli ($E^{\prime }$) and hardness (H) were obtained from 30 indentations separated from each other by 30 μm.

A dynamic mechanical analysis (DMA) was carried out on a Q800 analyzer (TA Instruments Inc., New Castle, Delaware, USA) to characterize the viscoelastic behavior of the substrates. During the sweep tests, the sample was clamped at both ends; a frequency of 1 Hz, a ramp rate of 5°C/min, a temperature range of 30 °C to 90 °C, and a force of 1 N were used. The instrument was completely calibrated in accordance with the procedures of TA Instruments. In these tests, $E^{\prime }$ is the storage modulus—i.e., the elastic component that measures the energy stored during one oscillation cycle—and is related to the sample stiffness; $E^{\prime \prime }$ is the loss modulus—i.e., the viscous component that measures the mechanical energy dissipated through molecular motion in an oscillation cycle; and tanδ is the relationship between the elastic and inelastic components (phase lag referred to as loss tangent) that arises from any of the several molecular-level loss processes such as entanglement, slip, or friction between the monomers. Furthermore, tanδ has higher values for amorphous polymers and lower values for more crystalline polymers [19,23].

The thermal behavior of the specimens manufactured in the pure and CuNi NW-loaded resin was characterized by differential scanning calorimetry (DSC) and thermogravimetric analysis (TGA) using an STA 449 F5 Jupiter system (NETZSCH, Selb, Germany) operated under a nitrogen atmosphere. Samples were analyzed between 30 and 600 °C with a heating rate of 10°C/min to determine their glass transition temperatures and crystallographic properties.

The Raman spectroscopy characterization of the substrates manufactured in pure and CuNi NW-loaded resin was performed to determine the vibration bands of the species on their surface. The Raman spectra were acquired using the Horiba Scientific confocal spectrometer LabRam HR (HORIBA, Kyoto, Japan) equipped with a 532 nm laser and a 100× microscope objective; all the Raman spectra were obtained with a 15 mW laser power and a grating of 600g/mm (slit aperture) for 8 s acquisition times. The LabSpec 6 software Horiba Scientific (HORIBA, Kyoto, Japan) was used for Raman spectra acquisition and analysis, where each sample was scanned in the range of $25–4000{\;\mathrm{cm}}^{-1}$.

The Fourier-transformed infrared (FTIR) spectra on substrates that were SLA-printed in pure and CuNi NW-loaded resin were obtained by the attenuated total reflection (ATR) technique in the Nicolet iS50 Spectrometer (Thermo Fisher Scientific, Waltham, MA, USA). The spectra were analyzed in terms of transmittance in the wavenumber range of $400–4000{\;\mathrm{cm}}^{-1}$, using a resolution of $4{\;\mathrm{cm}}^{-1}$ and an optical velocity of 0.1581cm/s; this analysis was used to determine the absorption bands of the organic functional groups on the surface of the substrates.

X-ray diffraction (XRD) patterns of the substrates in pure and CuNi NWs-loaded resin were acquired in the powder diffractometer Bruker model D8 Advance with DaVinci geometry under the following conditions: a 40 kV voltage, a 0.6 mm divergence gap, a LynxEye linear detector, a 0.02035° step, an acquisition time of 0.6 s per step, and a range of 3.5–70°. The qualitative analysis of the phases was performed through the comparison of the experimental XRD patterns with the database PDF-2 (2014) of the International Centre for Diffraction Data (ICDD).

## 3. Results and Discussion

### 3.1. Cost Considerations

The manufacturing time of a cylindrical specimen, with or without CuNi NWs, was approximately 340 s—i.e., 11 s per layer of $3.6{\;\mathrm{cm}}^{2}$. In the present case, considering only the chemicals used for the synthesis and washing of the NWs, the cost for the CuNi NWs reaches 0.45 €/mg, whereas it is 0.14 €/mL for the pure resin. Hence, the manufacturing cost of a functional device containing CuNi NWs greatly depends on their content. In this study, with a load of 0.36% w/w, the cost of each specimen of pure resin was approximately 0.14 €, while it was 1.65 € for those manufactured with CuNi NWs.

### 3.2. Morphology

**Figure 2:**
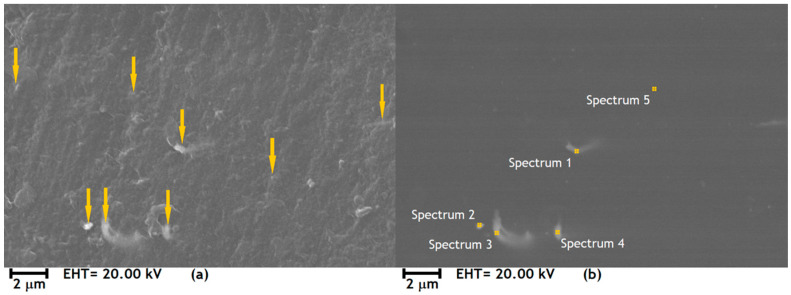
SEM images on the surface of a CuNi NW-loaded substrate; (**a**) secondary electron micrograph, with yellow arrows indicating the position of the CuNi NWs aggregates; and (**b**) backscattered electron micrograph, which shows the regions analyzed by EDS.

The micrograph in Figure 2a in secondary electron mode shows the topographical image of the surface of an SLA-printed, CuNi NW-loaded specimen, with yellow arrows pointing at CuNi NW aggregates embedded in the photopolymerized resin. These NW aggregates are even better highlighted in backscattered electron mode (Figure 2b) thanks to the large atomic number difference between Cu/Ni and the main elements in the resin, carbon (C) and oxygen (O) (the heavier the atomic number, the brighter the image). Hence, SEM observations evidence the presence of CuNi NW aggregates randomly dispersed in the polymeric matrix, which explains the absence of percolation.

EDS was also used on the surface the CuNi NW-loaded substrate to analyze its elemental composition. The results are summarized in Table 1 and the locations of the EDS analyses are shown on Figure 2b, with spectra 1 to 4 corresponding to CuNi NW aggregates and spectrum 5 to pure resin. In all the spectra, Cu and Ni correspond to the NWs, and C and O correspond to the resin. Cu and Ni are not detected in the region of spectrum 5 (pure resin), which means that the NWs were not degraded and dissolved in the polymeric matrix during the manufacturing process (at least not significantly, the EDS detection limit being 0.1 wt.%).

**Figure 3:**
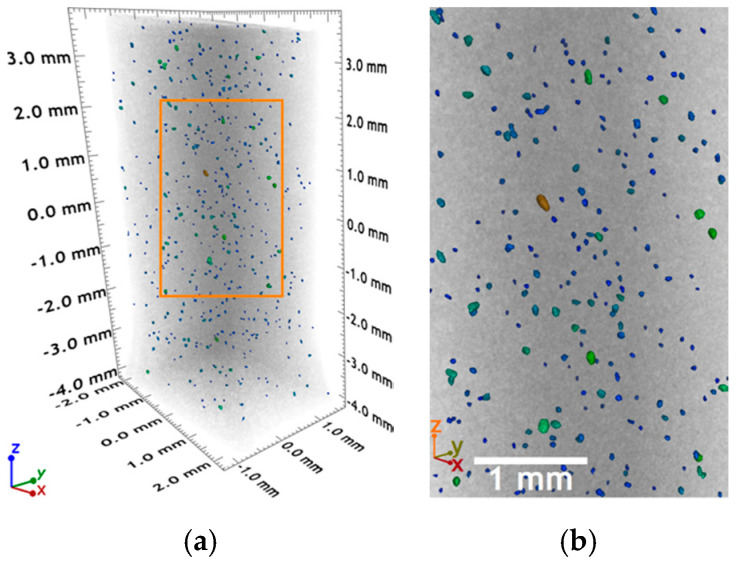
(**a**) 3D rendering of the CuNi NW-loaded resin and (**b**) magnification of the region in the red rectangle in (**a**).

In Figure 3, the X-ray tomogram shows the 3D distribution of the CuNi NW aggregates in the photopolymerized resin matrix after SLA printing. The brightness of an X-ray tomogram depends on the atomic number of the elements constituting the sample, with a higher atomic number giving a brighter image [19,24,25,26]. Given the large atomic number difference between Cu/Ni and the main elements constituting the resin (C and O), it was possible to map a random 3D distribution of the CuNi NWs in the polymeric matrix. In the analyzed region of $50.6{\;\mathrm{mm}}^{3}$, the detected volume of CuNi NWs approximately corresponds to 0.06 vol.%. Moreover, Figure 3 confirms the SEM results (Figure 2) at a 3D level. The CuNi NWs form randomly dispersed aggregates in the polymeric matrix, preventing their percolation.

To conclude on morphological considerations, due to randomly dispersed aggregates without percolation, the CuNi NWs are expected to influence the thermo-mechanical properties [19,27,28] of the polymer matrix but not its electrical properties [19,29]. The aggregation phenomenon is likely due to the resin’s high viscosity and the presence of polar groups on the polymeric chains [19,24,25,26]. In any case, a homogeneous dispersion of the NWs in the polymeric matrix without aggregates is the key to reach achieve percolation with a minimal NW content. Moreover, this would lead to an optimized manufacturability (closer to the pure resin), a lower cost (see Section 3.1), and homogeneous properties in the 3D-printed composites [19].

### 3.3. Mechanical and Thermal Analysis

**Figure 4:**
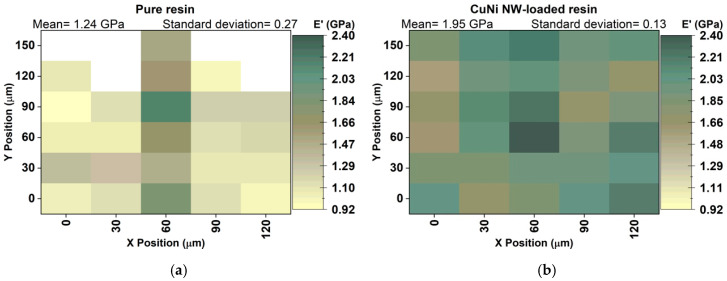

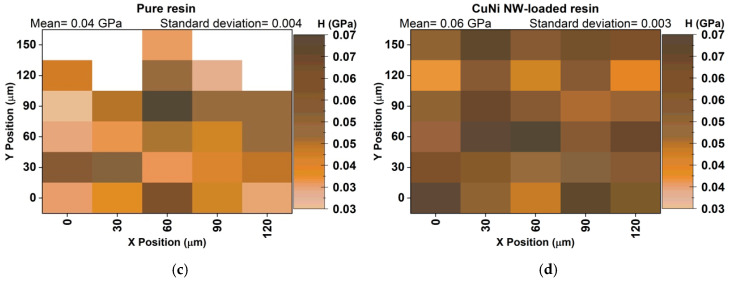
Mappings of the mechanical properties $E^{\prime }$ (**a**,**b**) and H (**c**,**d**), obtained from the nanoindentation tests on the surface of the pure and CuNi NW-loaded resin substrates, respectively. A total surface area of $120\times 150{\;\mu m}^{2}$ was analyzed on each substrate.

**Figure 5:**
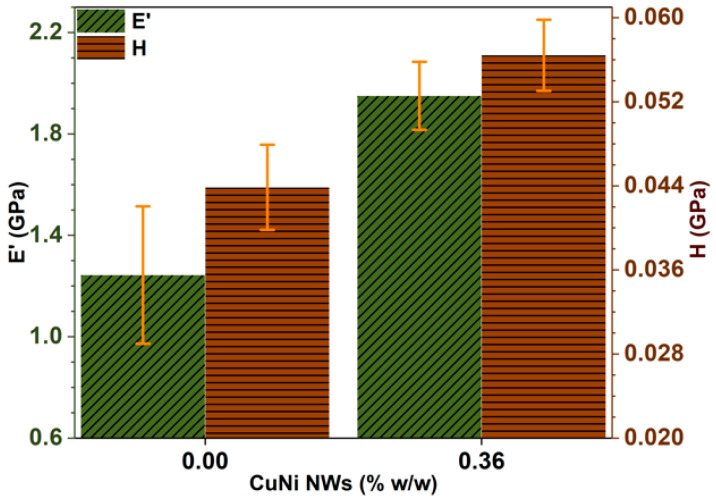
Average values of $E^{\prime }$ (green) and H (brown) for the pure and CuNi NW-loaded substrates, corresponding to the mappings in Figure 4.

Mappings of the mechanical properties $E^{\prime }$ (Figure 4a,b) and H (Figure 4c,d) were obtained from nanoindentation tests on the surfaces of pure and CuNi NW-loaded, SLA-printed substrates. The total surface area of the mappings is $120\times 150{\;\mu m}^{2},$ and the variations in the $E^{\prime }$ and H values are represented by color scales, with a darker color corresponding to a higher value. The advantage such mappings is to directly and visually assess local variations in the mechanical properties. Moreover, the average values extracted from these mappings can be representative of the entire specimens if the studied areas are large enough (Figure 5) [19,30,31].

In the case of the pure resin, the variations in $E^{\prime }$ and H are likely due to the SLA process itself (surface roughness, surface defects, etc.). In the case of the CuNi NW-loaded substrate, two other parameters influence $E^{\prime }$ and H: the NW aggregate concentration and the quality of the polymeric matrix/NW aggregate bonding. Since there is a significant increase in $E^{\prime }$ (57%) and H (50%) when loading the resin with NWs (Figure 5), it is assumed that the influence of the SLA process on $E^{\prime }$ and H can be neglected compared to the influence of the NWs. Moreover, as the dispersion of the NW aggregates seems random and rather uniform (Figure 2), it can be assumed that the main factor driving the variation in $E^{\prime }$ and H is the matrix/NW bonding.

The CuNi NW aggregates seem to act as hardeners inside the matrix, and a higher matrix/NW aggregate bonding quality provides a higher load transfer from the NWs to the matrix and, hence, higher mechanical properties ($E^{\prime }$ and H). On the contrary, some NW aggregates may be only slightly infiltrated by the polymer and constitute local defects with low mechanical properties. Obtaining well-dispersed fillers in a polymeric matrix (instead of aggregates) is consequently a major focus in many studies, as this is the key to obtain an optimized matrix/filler bonding, a maximal load transfer, and enhanced properties [19,30,31,32,33,34].

**Figure 6:**
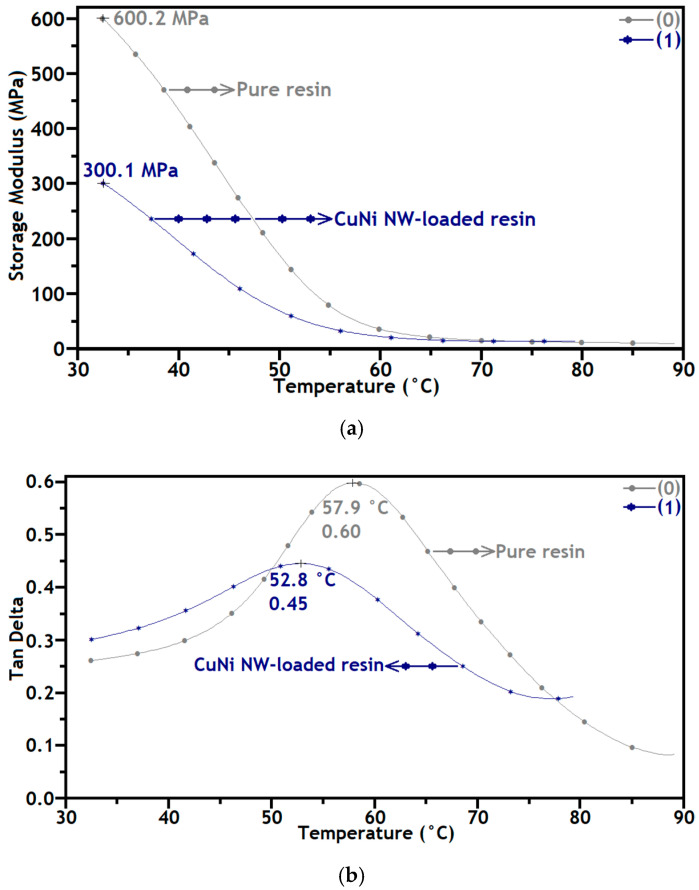
DMA curves of the pure (0) and CuNi NW-loaded (1) substrates for (**a**) $E^{\prime }$ and (**b**) tanδ, as a function of temperature.

Figure 6 presents the evolution of $E^{\prime }$ and tanδ as a function of the temperature, between 30 °C and 90 °C, for pure and CuNi NW-loaded substrates. Figure 6a shows that $E^{\prime }$ decreases for both substrates when the temperature increases between 30 °C and 60 °C, highlighting a glass transition region. Moreover, $E^{\prime }$ is significantly higher for the pure resin than for the CuNi NW-loaded one in this temperature range, and the maximum $E^{\prime }$ values, measured at 30 °C, are 600.2 and 300.1 MPa for the pure and CuNi NW-loaded substrates, respectively. Furthermore, factors such as the reaction degree and the crosslinking density mainly influence the value of $E^{\prime }$. The glass transition process is thereby confirmed, suggesting that the behavior of the material with and without CuNi NWs can be evaluated through the rheological properties.

In Figure 6b, the maximum values of tanδ correspond to the glass transition temperatures ($T_{g}$), 57.9 and 52.8 °C, of the pure and CuNi NW-loaded resins, respectively (see also Table 2) [35]. Loading the polymeric matrix with CuNi NWs lowers the $T_{g}$, which could be due to a poor resin matrix/NW aggregate bonding. Indeed, a lack of chemical interaction between the resin and the CuNi NWs can decrease the crosslinking density and lead to a higher mobility of the polymeric chains in the composite, resulting in a lower $T_{g}$ [19,35,36,37,38,39]; this is confirmed by a sharper peak around the maximum value of tanδ for the pure resin, which indicates a more ordered structure due to a higher crosslinking density.

The addition of CuNi NWs highly influenced the molecular dynamics in the polymeric matrix, which resulted in a reduction in $T_{g}$ and $E^{\prime }$ [35,36]. The DMA results show a more elastic behavior of the CuNi NW-loaded substrate compared to the pure resin one, suggesting the enhanced stiffness of the material.

**Figure 7:**
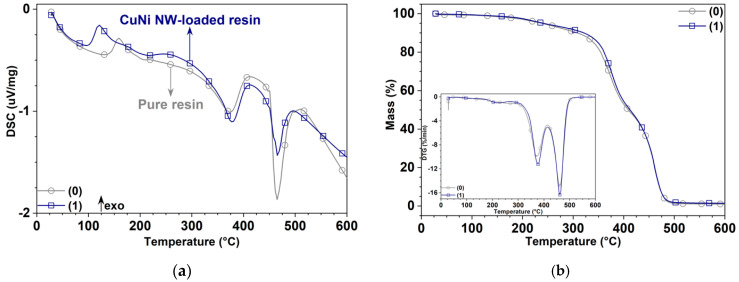
Thermal analysis of the pure (0) and CuNi NW-loaded (1) substrates by (**a**) DSC, (**b**) TGA, and DTGA (insert) as a function of the temperature.

The DSC curves in Figure 7a show a broad glass transition region between 32 and 135 °C for the pure substrate ($T_{g}=60.8\;{}^{\circ}C$) and between 31 and 97 °C for the CuNi NW-loaded one ($T_{g}=51.6\;{}^{\circ}C$) [35]; thus, the glass transition region and $T_{g}$ slightly decreased when loading the resin with CuNi NWs, and the results were in agreement with the DMA analysis (see Table 2). Moreover, the crystallization temperature, corresponding to the maximum of the first exothermal peak, is lower for the CuNi NW-loaded composite (ca. 121 °C) than for the pure resin (ca. 160 °C).

The thermal stability of the samples was investigated by TGA between 32 and 600 °C under an $N_{2}$ atmosphere (Figure 7b); the slight mass loss between 80 and 100 °C may be due to the evaporation of adsorbed IPA in the resin, although most of it should have evaporated during the manufacturing process. The weight loss between 100 and 300 °C was mainly attributed to the evaporation of physiosorbed and chemisorbed water [35]. Finally, most of the thermal degradation of the samples occurring between 300 and 500 °C was likely due to the evaporation of organic species from the resin (e.g., methacrylic acid and ester) [19,35,36,37,38,39]. Moreover, as the TGA curves are similar for both materials, it can be assumed that the NWs were not significantly degraded during the heating ramp and that the weight loss was mainly driven by the polymeric matrix degradation.

A mechanical and thermal analysis of pure and CuNi NW-loaded, SLA-printed samples was carried out. It seems that the addition of NWs to the polymeric matrix increased the mechanical surface properties (Figure 4 and Figure 5) thanks to a load transfer phenomenon. However, it decreased the thermomechanical bulk properties (Figure 6 and Figure 7) due to interfacial defects in the composite material (weak NW/matrix bonding).

### 3.4. Spectroscopic Characterization

**Figure 8:**
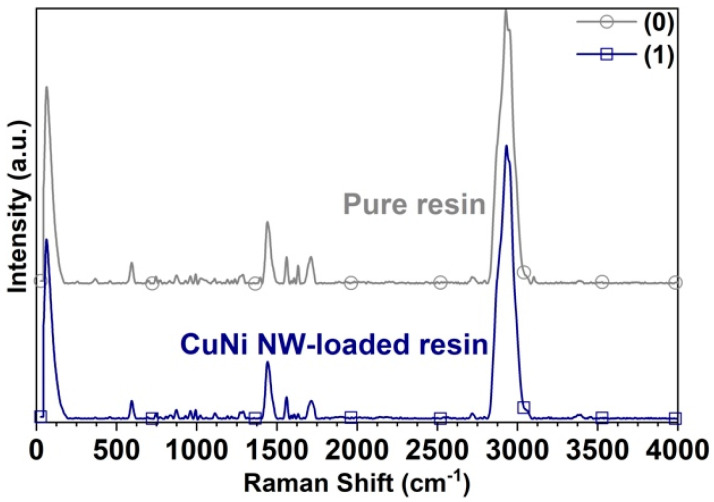
Raman spectra acquired on the substrates printed with (0) pure resin and (1) CuNi NW-loaded resin.

The Raman spectra obtained on the substrates manufactured in the “Clear FLGPCL 02” pure resin and CuNi NW-loaded resin at 0.36% w/w are presented in Figure 8, where Cu and Ni present vibrational modes at very low frequencies (phonon frequencies, $\sim 10{\;\mathrm{cm}}^{-1}$).

The spectrum of Figure 8 from the surface of the pure resin substrate reveals three intense Raman peaks at 1441 (strong), 2928, and $2948{\;\mathrm{cm}}^{-1}$ (very intense) that correspond to the C−H bond vibrations. Consequently, the medium and weak peaks at 1560 and $1590{\;\mathrm{cm}}^{-1}$ are attributed to the C−N−H and $C-{\mathrm{NO}}_{2}$ bond vibrations, respectively. The peaks at $595{\;\mathrm{cm}}^{-1}$ (medium) and $1608{\;\mathrm{cm}}^{-1}$ (weak) correspond to the C−C and C=C bond vibrations, respectively. The medium peaks at 1632 and $1712{\;\mathrm{cm}}^{-1}$ refer to the C=O symmetric and antisymmetric bond vibrations, and the one at $1396{\;\mathrm{cm}}^{-1}$ to the ${\mathrm{CH}}_{3}$ asymmetric bond stretching vibration. The band between $785^{}$ and $980{\;\mathrm{cm}}^{-1}$ corresponds to the C−O−C bond vibrations, the one between $1000^{}$ and $1160{\;\mathrm{cm}}^{-1}$ to the C−O−C bond asymmetric stretching vibrations, and the one between $1300^{}$ and $1380{\;\mathrm{cm}}^{-1}$ to the $C-{\mathrm{CH}}_{3}$ bond vibrations. Additionally, the weak peaks at 2650–2810 and $3050–3500{\;\mathrm{cm}}^{-1}$ are attributed to the O−C−H and O−H bond vibrations, respectively.

Raman spectroscopy reveals the surface interaction between organic materials and metallic nanostructures through surface-enhanced Raman scattering. Figure 8 shows similar Raman spectra from the pure resin and CuNi NW-loaded resins; however, it is possible to observe intensification in vibrational signals corresponding to the C−H ($2928{\;\mathrm{cm}}^{-1}–2948{\;\mathrm{cm}}^{-1}$) and O−H ($3050{\;\mathrm{cm}}^{-1}–3500{\;\mathrm{cm}}^{-1}$) vibrational modes [19,35,40,41,42]. This is evidence of the surface interaction between the polymeric resin and CuNi NWs through the C−H and O−H functional groups. Furthermore, metallic structures such as Cu and Ni do not present vibrational frequencies (phonon frequencies) in the range of wavenumbers observed in this study. Thus, this surface interaction can be responsible for the difference in $T_{g}$ before and after doping. Additionally, these studies allowed us to identify differences in the degree of crystallinity due to the influence of the doping agents.

The characteristic Raman spectrum of the photopolymer resin matrix shows a main peak with a maximum intensity of 1780 counts in the pure resin and 1834 counts in the one with nanowires; the peak also exhibits a wavelength shift from 2926 to $2930{\;\mathrm{cm}}^{-1}$ with the addition of the nanofillers. The increase in the intensification of the peak with nanowires corresponds to the energy difference in the vibrational state of the methacrylate molecule, which is related both to the level of polymer structuring and to electromagnetic intensification due to the plasmonic effect produced by the presence of the nanowires in the polymer matrix. On the other hand, the displacement of the peak to a greater wavelength indicates a change in the electronic dispersion of the molecules on the surface of the polymer, which implies evident structural changes in the molecular bonds and a greater crosslinking that corroborate the thermo-mechanical results shown.

**Figure 9:**
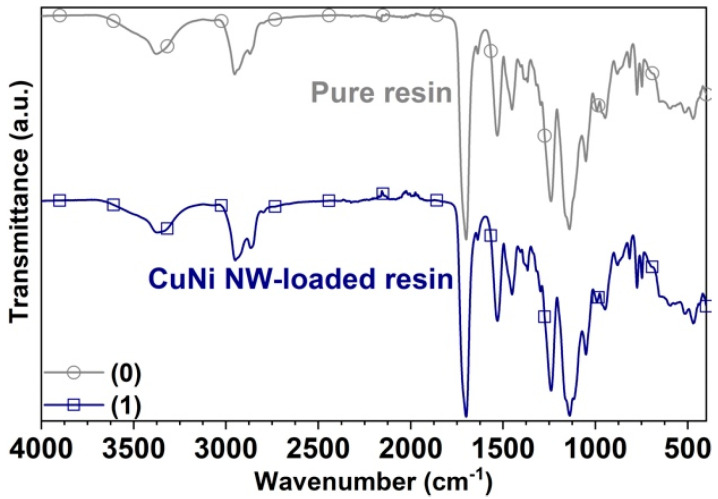
FTIR spectra acquired on the substrates printed with (0) pure resin and (1) CuNi NW-loaded resin.

In order to determine if the increasing aggregate of nickel-copper nanowires affects the chemical structure of the photopolymer resin, FTIR measurements were conducted to determine whether alterations occur in the main chemical groups of the polymer. The results show no alterations attributable to the presence of the CuNi NWs in the concentration studied —0.36% w/w; therefore, the polymer adequately fulfills its support function as a nanomaterial (Figure 9).

Figure 9 presents the FT-IR spectrum of the substrates manufactured in pure resin and CuNi NW-loaded resin, where there are distinct absorption bands from 1150 to $1250{\;\mathrm{cm}}^{-1}$, which can be attributed to the C−O−C stretching vibrations. The two bands at 1386 and $748{\;\mathrm{cm}}^{-1}$ can be attributed to the α-methyl group vibrations. Moreover, the bands at 865, 1297, 1367, and $1405{\;\mathrm{cm}}^{-1}$ can be attributed to the C−C, $O-{\mathrm{CH}}_{2}$, C−H, and C−OH stretching vibrations, respectively. The band at $1700{\;\mathrm{cm}}^{-1}$ indicates the presence of the ester group C=O stretching vibrations. The band at $1451{\;\mathrm{cm}}^{-1}$ can be attributed to the bending vibrations of the C−H bonds of the $-{\mathrm{CH}}_{3}$ groups. The three bands at 2952, 2932, and $2862{\;\mathrm{cm}}^{-1}$ can be assigned to the C−H bond stretching vibrations. Furthermore, there are two weak absorption bands at 3375 and $1637{\;\mathrm{cm}}^{-1}$, which can be attributed to the −OH group stretching and bending vibrations, respectively. Notwithstanding this, there are no appreciable differences between the FTIR spectra of the pure resin and the CuNi NW-loaded resin because the resin does not form any covalent bond with this type of nano-additives; additionally, the nanowires do not add new functional groups, which can be observed with infrared. According to the above discussions, it can be concluded that the photoreactive polymer resin is composed of a macromolecular methacrylic acid (MAA) and a methyl methacrylate (MMA) mixture [19,35,39,43,44].

**Figure 10:**
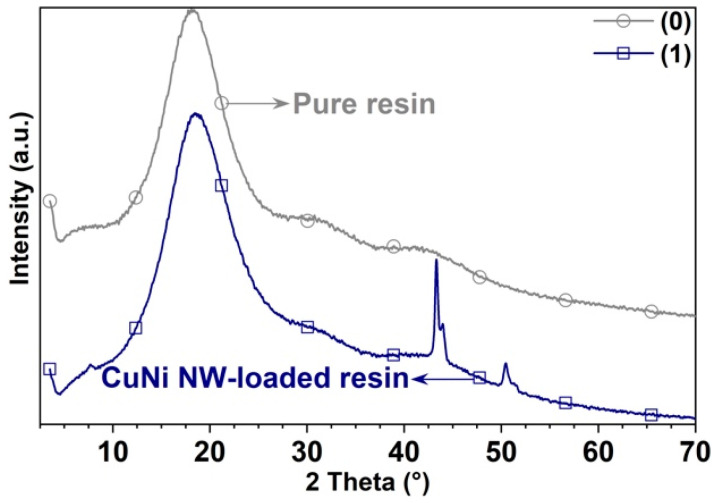
XRD patterns acquired on the substrates printed with (0) pure resin and (1) CuNi NW-loaded resin.

The semiquantitative analysis of the phases found in the specimens was performed using the Rietveld method. The percentages determined correspond to the polycrystalline phases without considering the percentage of amorphous material. The XRD patterns of Figure 10 prove that the CuNi NWs contained in some of the substrates manufactured by 3D printing have a face-centered cubic structure (in agreement with the database PDF-02 of the ICDD). Additionally, the contents of 59.8% copper and 40.2% nickel were evaluated in a semiquantitative analysis. No diffraction peak was observed on the XRD pattern of the “Clear FLGPCL 02” resin without nanowires because it is amorphous.

The spectroscopy analysis clearly indicates some structural changes, which might be due to an intermolecular transformation; thus, the incorporation of the nanowires affects the polymerization process, decreasing the scattering and crosslinking points. The results obtained allowed us to know the morphologic, mechanical, thermal, and spectrometric behavior of photopolymers resins, with and without CuNi NWs, used for 3D printing by the SLA technique. Notwithstanding this, the effect of CuNi NWs was not as predominant as expected. The reason for this is that many parameters involved in the manufacturing process, such as the concentration and dispersion of the CuNi NWs, the specimens’ size, and the polymerizing process interaction between the laser of the printer and the CuNi NWs, caused the physicochemical properties to not improve significantly with regard to the pure resin. Moreover, the highlight of this research was that specimens of cylindrical geometry reinforced with CuNi NWs were successfully manufactured by 3D printing through the SLA technique.

## 4. Conclusions

This paper presents the manufacturing of new polymeric resin substrates functionalized with CuNi NWs by the SLA technique. Copper-nickel alloy nanowires were successfully dispersed in the “Clear FLGPCL 02” resin, which is a typical resin used in 3D printing by the stereolithography technique. Besides the functionalization improvements, the manufacturability of three-dimensional objects by the SLA technique (3D printing) using a mass-functionalized photopolymer with CuNi NWs is demonstrated. Moreover, a homogeneous 3D distribution of the CuNi NWs inside the polymeric matrix was achieved, and the presence of the nanowires correlated with the modification of the mechanical and thermal properties of the resin. It was also demonstrated that the addition of CuNi NWs reduces the crosslinking density and leads to the higher mobility of the polymeric chains during the glass transition. Furthermore, both the melting and degradation temperatures remained unchanged below 600 °C, which was expected as Cu and Ni have higher melting points (1085 and 1455 °C, respectively). This study underscores the possible use of the SLA technique, in combination with functional nanomaterials, in the fabrication of new precision devices and for wearable electronics such as advanced transducers, flexible sensors, circuit/devices using self-assembly approaches, robotic parts, microfluidic devices, and microelectromechanical systems.

## Figures and Tables

**Table 1:** Atomic contents (in %) of the elements detected by EDS on the surface of a CuNi NW-loaded substrate.

Element	Spectrum (At %)				
	1	2	3	4	5
C k	83.6	85.5	84.3	84.0	87.3
O k	14.9	12.7	14.2	9.1	12.7
Cu k	1.0	1.4	1.0	5.7	-
Ni k	0.5	0.4	0.5	1.2	-

**Table 2:** Values determined from the DMA, DSC, and TGA analysis.

CuNi NWs Content	Transition and Degradation Temperatures (°C)	
	$T_{g}-DMA$	$T_{g}-DSC$
0.00% w/w	57.9	60.8
0.36% w/w	52.8	51.6
